# Low tumour-infiltrating lymphocyte density in primary and recurrent glioblastoma

**DOI:** 10.18632/oncotarget.28069

**Published:** 2021-10-12

**Authors:** Kelsey Maddison, Moira C. Graves, Nikola A. Bowden, Michael Fay, Ricardo E. Vilain, Sam Faulkner, Paul A. Tooney

**Affiliations:** ^1^School of Biomedical Sciences and Pharmacy, The University of Newcastle, Callaghan, NSW, Australia; ^2^School of Medicine and Public Health, The University of Newcastle, Callaghan, NSW, Australia; ^3^Hunter Cancer Biobank, The University of Newcastle, Callaghan, NSW, Australia; ^4^Centre for Drug Repurposing and Medicines Research, The University of Newcastle, Callaghan, NSW, Australia; ^5^Hunter Medical Research Institute, New Lambton Heights, NSW, Australia; ^6^GenesisCare, Lake Macquarie Private Hospital, Gateshead, NSW, Australia; ^7^Pathology North, Hunter New England Area Health Service, New Lambton Heights, NSW, Australia

**Keywords:** glioblastoma, tumour-infiltrating lymphocytes, programmed cell death 1, tumour recurrence, immune microenvironment

## Abstract

Immunotherapies targeting tumour-infiltrating lymphocytes (TILs) that express the immune checkpoint molecule programmed cell death-1 (PD-1) have shown promise in preclinical glioblastoma models but have had limited success in clinical trials. To assess when glioblastoma is most likely to benefit from immune checkpoint inhibitors we determined the density of TILs in primary and recurrent glioblastoma. Thirteen cases of matched primary and recurrent glioblastoma tissue were immunohistochemically labelled for CD3, CD8, CD4 and PD-1, and TIL density assessed. CD3+ TILs were observed in all cases, with the majority of both primary (69.2%) and recurrent (61.5%) tumours having low density of TILs present. CD8+ TILs were observed at higher densities than CD4+ TILs in both tumour groups. PD-1+ TILs were sparse and present in only 25% of primary and 50% of recurrent tumours. Quantitative analysis of TILs demonstrated significantly higher CD8+ TIL density at recurrence (*p* = 0.040). No difference was observed in CD3+ (*p* = 0.191), CD4+ (*p* = 0.607) and PD-1+ (*p* = 0.070) TIL density between primary and recurrent groups. This study shows that TILs are present at low densities in both primary and recurrent glioblastoma. Furthermore, PD-1+ TILs were frequently absent, which may provide evidence as to why anti-PD-1 immunotherapy trials have been largely unsuccessful in glioblastoma.

## INTRODUCTION

Glioblastoma, the most common and aggressive brain tumour occurring in adults, accounts for ~56% of newly diagnosed central nervous system (CNS) cancers in Australia [1]. Standard treatment involves maximal safe surgical resection, followed by radiation therapy with concomitant and adjuvant temozolomide chemotherapy. In most cases, a treatment resistant recurrent tumour occurs, and median survival time remains short at 14.6 months [2].

Tumour-infiltrating lymphocytes (TILs) have been reported in a number of cancers. The immune checkpoint molecule, programmed cell death 1 (PD-1), is expressed by activated T cells and regulates immune responses by binding its ligand, programmed cell death ligand 1 (PD-L1) [3], which can be expressed by tumour cells [4–9]. Sustained antigen presentation and the binding of PD-L1 by TILs expressing PD-1 can result in T cell exhaustion and tumour evasion of immune responses [3, 10]. Immune checkpoint inhibition with anti-PD-1 antibodies blocks PD-1/PD-L1 interactions, restoring effector T cell proliferation and function [11]. This approach has been successful in some cancers including melanoma, where increased progression-free and overall survival, and higher response rates than treatment with chemotherapy have been observed [12]. In melanoma, response to anti-PD-1 immunotherapy has been associated with higher density of CD8+ (and PD-1+) TILs [11].

Until recently, the brain was considered an “immune privileged” site. However, peripheral antigen presenting cells and T cells have been reported in various locations in the CNS [13–15]. Dendritic cells have been identified among the antigen presenting cells that migrate to cervical lymph nodes, and are believed to contribute to CNS immunity and tolerance through antigen presentation to T cells at this site [15]. Under normal conditions, T cells are able to migrate to perivascular spaces within the brain and, in various disease states, antigen-specific T cells undergo a second stage of migration across the astrocytic endfeet barrier into the brain parenchyma [16].

TILs have been observed in glioblastoma, though findings in relation to their prognostic value have been inconsistent [6, 17–20]. While a number of studies report the presence of TILs and/or detection of PD-1 and PD-L1 in immunohistochemical investigations of tumour tissue [6–9], these focus largely on primary glioblastoma, with very few comparisons to matched recurrent tumour tissue. As PD-1 presents a potential target for immunotherapy in glioblastoma, further research is warranted in order to clarify these inconsistencies.

Previous clinical trials conducted using anti-PD-1 immunotherapy in glioblastoma have demonstrated limited success [21, 22]. For example, the recent CheckMate 143 trial found no significant overall survival benefit in recurrent glioblastoma with anti-PD-1 when compared to the anti-angiogenic drug bevacizumab [23]. Another study reported similar anti-tumour activity when anti-PD-1 plus bevacizumab was compared to bevacizumab alone [24]. However, a recent study by Cloughesy et al (2019) [25] found that anti-PD-1 therapy given before and after recurrent tumour resection was associated with significant increases in overall survival compared to anti-PD-1 therapy given only after recurrent surgery. In contrast to prior studies assessing TILs in primary tumour tissue, the majority of clinical studies have assessed response to immunotherapy in recurrent glioblastoma. Given these discrepencies, and the finding by Cloughesy et al (2019) [25] of increased overall survival for patients treated with neoadjuvant anti-PD-1 at recurrence, we aimed to determine whether CD3+ TILs overall, or CD4+, CD8+ or PD-1+ TIL subsets, differed between matched samples of primary and recurrent glioblastoma and assess when anti-PD1 immunotherapy would be of most benefit.

## RESULTS

### Categorical scoring

**Figure 1:**
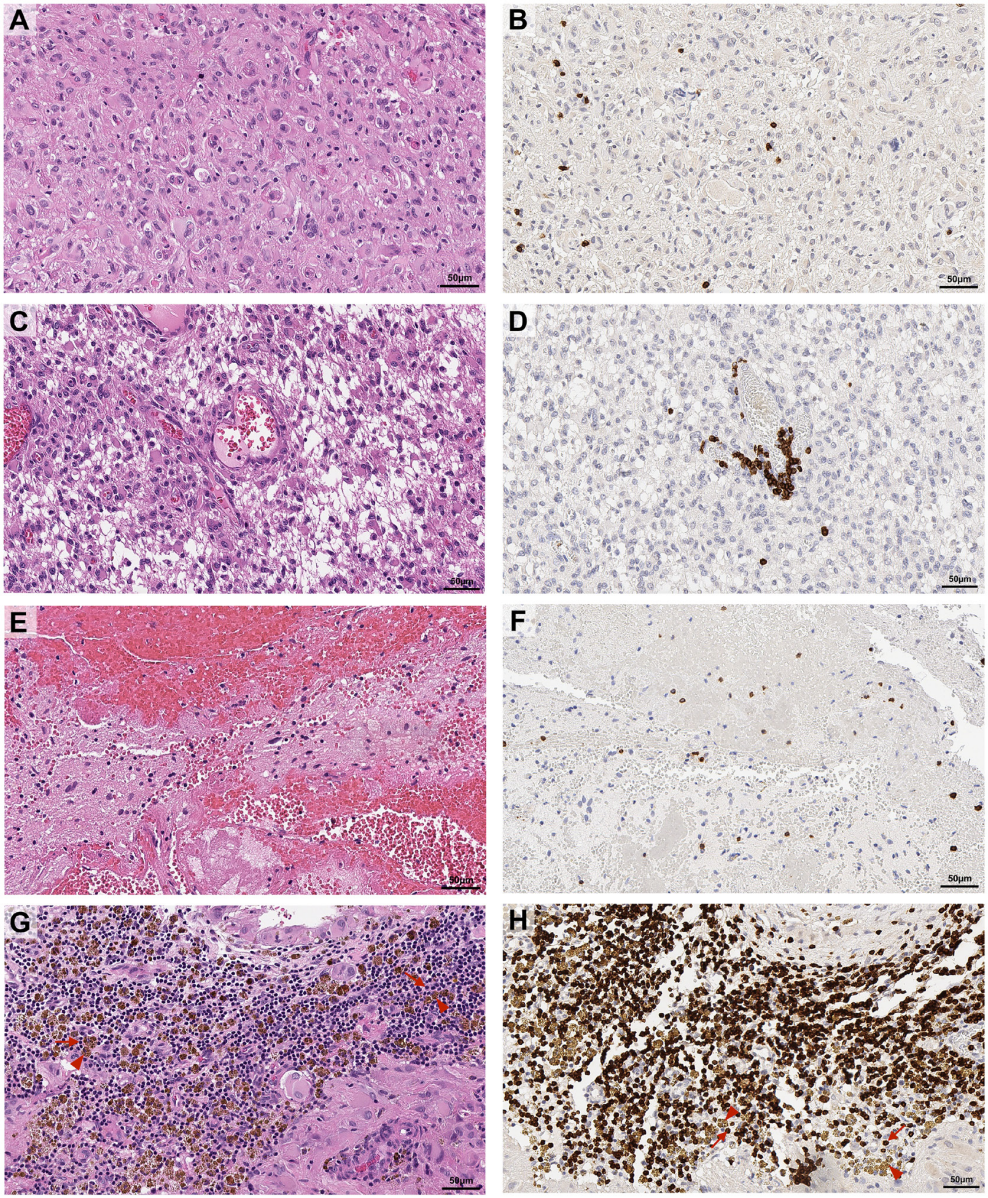
Representative images of topographical localisation of T cells in glioblastoma tumours. Glioblastoma sections were stained with H&E (**A**, **C**, **E**, **G**) or labelled for CD3+ TILs by DAB immunohistochemistry (**B**, **D**, **F**, **H**). CD3+ TILs were located in the tumour proper (A, B), in perivascular spaces (C, D), associated with bleeds with prominent erythrocytes (E, F) and associated with haemosiderin deposits (G, H). Red arrows indicate TILs, red arrow heads indicate bronze haemosiderin deposits (G, H). Scale bar = 50 μm.

**Figure 2:**
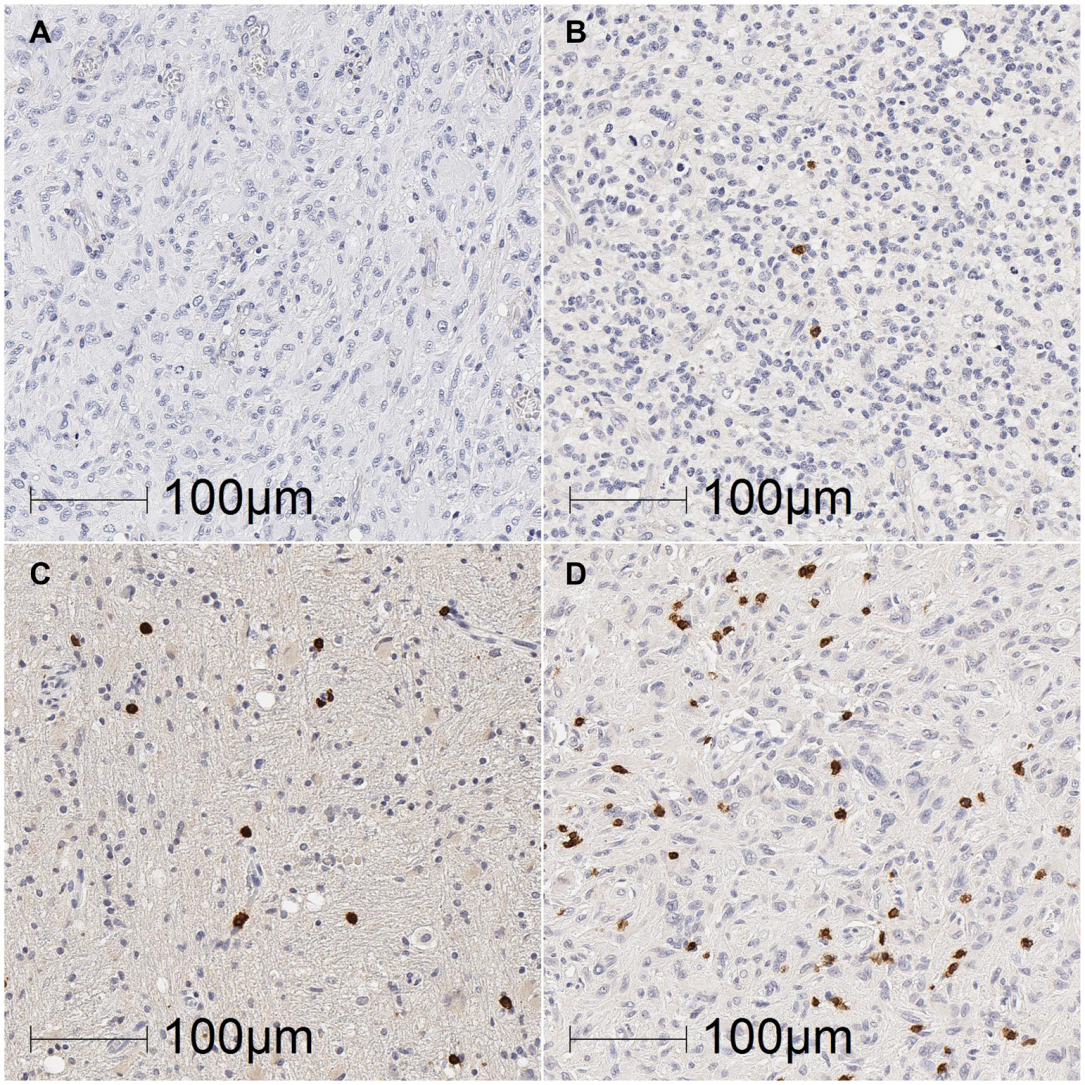
Representative images of TIL density in glioblastoma tumours. Glioblastoma sections were labelled for CD3+, CD8+, CD4+ and PD-1+ TILs by DAB immunohistochemistry and scored based on the density of TILs as 0 (absent; **A**), 1 (present; **B**), 2 (moderate; **C**), or 3 (marked; **D**). Representative images from sections labelled for CD3+ TILs (B, C and D) or CD4+ TILs (A; no sections labelled for CD3 had completely absent TILs). Scale bar = 100 μm.

Assessment of full-face tumour sections allowed for the observation of T cell presence and density across various topographies within each tumour (Figure 1). The overall level of TILs in glioblastoma tumours was very low. Only one tumour demonstrated marked CD3+ TIL density (a score of 3) relative to other glioblastomas (Figure 2 and Table 1), whilst the majority of both primary and recurrent tumours scored a 1, suggesting T cell infiltration into the tumour proper was low but not completely absent (Figure 2 and Table 1). CD8+ TILs were the dominant T cell subset present in the tumour tissue, demonstrating similar density to CD3+ TILs overall within the tumour proper. CD4+ TILs were observed less frequently and PD-1+ TILs were often completely absent (Table 1).

**Table 1:** Categorical density scores for CD3+, CD8+, CD4+, and PD-1+ TILs

	Primary				Recurrence			
	0	1	2	3	0	1	2	3
**CD3**								
Tumor	-	9 (69.2%)	4 (30.8%)	-	-	8 (61.5%)	4 (30.8%)	1 (7.7%)
Perivascular	2 (15.4%)	8 (61.5%)	2 (15.4%)	1 (7.7%)	2 (15.4%)	7 (53.8%)	4 (30.8%)	-
Bleeds	7 (53.8%)	5 (38.5%)	-	1 (7.7%)	6 (46.2%)	4 (30.8%)	-	3 (23.1%)
**CD8**								
Tumor	-	9 (69.2%)	4 (30.8%)	-	-	8 (61.5%)	4 (30.8%)	1 (7.7%)
Perivascular	5 (38.5%)	6 (46.2%)	2 (15.4%)	-	2 (15.4%)	8 (61.5%)	3 (23.1%)	-
Bleeds	8 (61.5%)	4 (30.8%)	-	1 (7.7%)	5 (38.5%)	5 (38.5%)	1 (7.7%)	2 (15.4%)
**CD4**								
Tumor	9 (69.2%)	3 (23.1%)	1 (7.7%)	-	3 (23.1%)	9 (69.2%)	1 (7.7%)	-
Perivascular	5 (38.5%)	5 (38.5%)	2 (15.4%)	1 (7.7%)	3 (23.1%)	9 (69.2%)	1 (7.7%)	-
Bleeds	9 (69.2%)	3 (23.1%)	-	1 (7.7%)	6 (46.2%)	6 (46.2%)	1 (7.7%)	-
**PD-1**								
Tumor	9 (75.0%)	3 (25.0%)	-	-	6 (50.0%)	4 (41.6%)	1 (8.3%)	-
Perivascular	10 (83.3%)	2 (16.7%)	-	-	9 (75.0%)	3 (25.0%)	-	-
Bleeds	12 (100.0%)	-	-	-	10 (83.3%)	2 (16.7%)	-	-

Similar to the tumour proper, perivascular CD3+ T cell density was predominantly low and similar between primary and recurrence (Table 1). The density of CD8+ T cells in perivascular spaces was higher at recurrence with 84.6% of cases having a score ≥ 1, compared to 61.5% of primary tumours (Table 1). CD4+ T cells were predominantly scored ≤ 1 in perivascular spaces in both primary (76.9%) and recurrent (92.3%) tumours. Again, PD-1+ T cells were virtually absent in the perivascular spaces of the majority of tumours (Table 1).

Signs of bleeding or previous haemorrhage included red blood cells within the tumour tissue or haemosiderin deposits. T cells in close proximity to these features were not considered to have infiltrated the tumour. Interestingly, in tumours with prominent haemosiderin deposits, these were associated with marked density of CD3+ T cells, and T cells in this location accounted for the majority of T cells observed in these cases (Figure 1 and Table 1). CD8+ T cells associated with bleeds followed a similar pattern, while CD4+ T cells were present at lower densities. PD-1+ T cells were not present in this topographical location in any primary tumours and present in only 16.7% of recurrent tumours in this location (Table 1).

### Quantitative scoring

**Figure 3:**
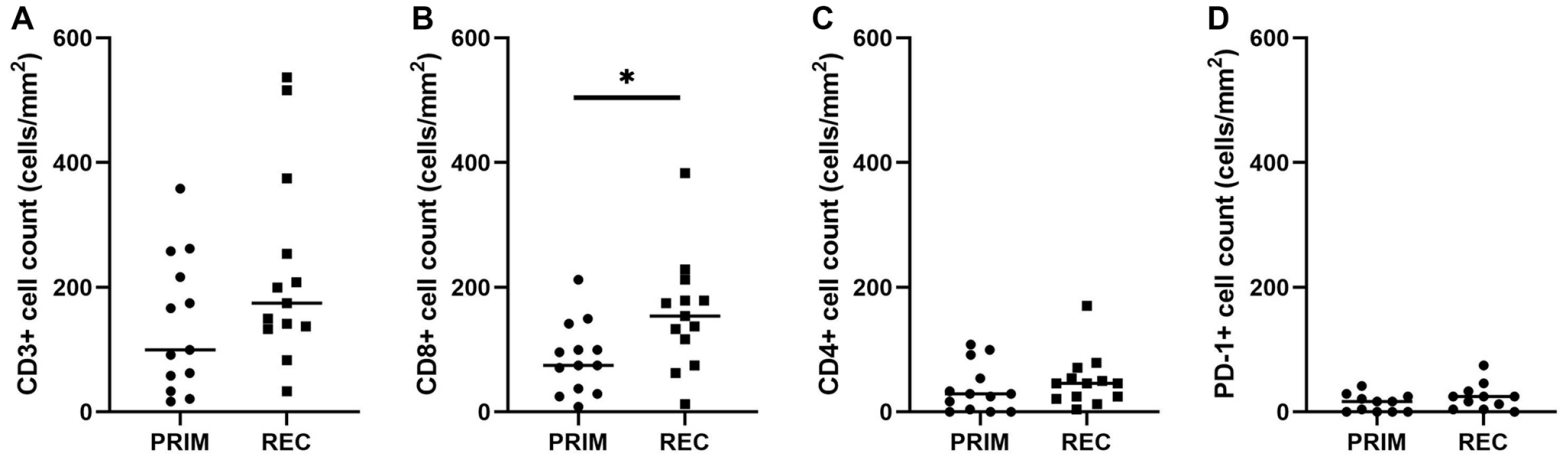
Quantitative TIL scores in primary and recurrent glioblastoma. Density of CD3+, CD8+, CD4+ and PD-1+ TILs was calculated for each case and compared between primary and recurrent groups. No difference was seen in overall CD3+ TIL density (*p* = 0.191; **A**) CD8+ TILs were present at significantly higher density in recurrent tumours compared to primary tumours (*p* = 0.040; **B**). No differences were seen in CD4+ TIL density (*p* = 0.607; **C**) or PD-1+ TIL density (*p* = 0.070; **D**). Bars represent median score. PRIM: primary tumour, REC: recurrent tumour.

There were significant correlations between categorical and quantitative scoring methods for all T cell markers (Supplementary Table 1). There was no significant difference (*p* = 0.191) in CD3+ cell counts in the primary (*n* = 13, mean 139.88 ± 108.60 cells/mm^2^) compared to recurrent tumours (*n* = 13, 226.30 ± 156.55 cells/mm^2^) (Figure 3A). Within T cell subsets, CD8+ TIL counts increased significantly (*p* = 0.040) in the recurrent tumours (*n* = 13, 157.48 ± 91.13 cells/mm^2^) compared to the primary tumours (*n* = 13, 86.10 ± 57.29 cells/mm^2^) (Figure 3B) with 9 of 13 cases showing higher CD8+ TILs at recurrence (Supplementary Figure 1). However, the CD4+ cell counts did not differ (*p* = 0.607) between primary (*n* = 13, 37.77 ± 38.90 cells/mm^2^) and recurrent groups (*n* = 13, 49.93 ± 42.33 cells/mm^2^) (Figure 3C). Whilst PD-1+ TIL density also increased slightly in recurrent (*n* = 11, 24.21 ± 21.69) compared to primary (*n* = 11, 14 ± 14.33) tumours, this trend was not significant (*p* = 0.070) (Figure 3D).

## DISCUSSION

Anti-PD-1 immunotherapy has been proposed as a potential treatment option for glioblastoma. Despite preclinical studies suggesting promising results from anti-PD-1 alone and in combination with other treatments [26–28], clinical trials and retrospective studies assessing the efficacy of this treatment in recurrent tumours have shown limited improvement in survival for patients [23, 24, 29, 30]. Therefore, we assessed T cell populations in matched primary and recurrent tumours, and determined whether changes occurred in these populations over the course of the disease. Importantly we examined PD-1+ T cell density to inform future studies assessing the suitability of anti-PD-1 immunotherapies in treatment of glioblastoma.

Categorical scoring for the T cell markers CD3, CD8, CD4 and PD-1 indicated that the density of TILs across both primary and recurrent glioblastoma tumours was low. It has been suggested that immunosuppression reported in glioblastoma is due to treatments, including radiation and temozolomide [31–33]. Additionally, patients are often treated with dexamethasone, a corticosteroid used to relieve the symptoms associated with oedema caused by the tumour [33, 34] but its immunosuppressive effects may reduce anti-tumour immunity [33, 35, 36]. However, the majority of cases in this cohort were considered not affected by dexamethasone or temozolomide at the time of surgery as the last reported dose was given more than 28 days prior to surgery. While the time since last dexamethasone dose and the amount given may differ between patients, only three cases in our cohort were known to have received dexamethasone at any dose within 28 days of surgery. Low TIL density across the cohort was therefore considered to be unlikely due to dexamethasone or temozolomide treatment. A previous study by Berghoff et al (2015) [6] yielded similar results, as CD3+ T cells were reported in the majority (66.7%) of primary tumour sections assessed, but were present most frequently at low density. This study also reported no correlation between dexamethasone treatment prior to surgery and density of CD3+ TILs [6]. Alternatively, the low density of TILs observed in glioblastoma may result from the sequestration of naïve T cells in the bone marrow, as suggested by Chongsathidkiet et al (2018) [37]. However, further assessment of peripheral T cell populations is required to determine the extent to which this may contribute to lack of TILs in glioblastoma.

Both the categorical and quantitative scoring suggested that TILs were predominantly cytotoxic CD8+ T cells, with CD4+ T cells present less frequently and at lower densities across the cohort. Quantification of TILs revealed a significant increase in CD8+ T cells in recurrent compared to primary tumours. Tumeh et al (2014) [11] reported that a higher baseline density of CD8+ TILs was associated with response to anti-PD-1 immunotherapy in melanoma. However, the density of CD8+ TILs in patients that responded to anti-PD1 immunotherapy ranged from ~500–8000 cells/mm^2^ in that study [11]. In contrast, we observed a mean density of 157.48 ± 91.13 CD8+ cells/mm^2^ in recurrent glioblastoma. Furthermore, whilst a recent study by Klemm et al (2020) [38] using flow cytometry of tumour tissue showed higher levels of lymphocytes in glioblastoma compared to normal tissue, this level was still significantly less than that observed from melanoma brain metastases. Therefore, despite being statistically significant, the increase in CD8+ TILs that we observed in recurrent glioblastoma may not be enough to induce a biologically or clinically significant response to immunotherapy. We also acknowledge that small sample size is a limitation of our study, and whether an increase in CD8+ TILs is observed in a larger cohort of matched patient samples is yet to be confirmed.

One study by Cloughesy et al (2019) [25] noted a significant increase in overall survival in 16 patients given anti-PD-1 immunotherapy before and after surgery to remove a recurrent glioblastoma, compared to 16 patients with a recurrence that received anti-PD-1 immunotherapy only after surgery. Whilst this study did not find a significant difference in CD8+ TILs between the two patient groups, some but not all patients given anti-PD-1 immunotherapy before and after recurrent surgery had higher CD8+ cells compared to those patients given adjuvent therapy alone. Whether the significant increase in CD8+ TILs at recurrence in our cohort provides support for the study by Cloughesy et al (2019) needs to be interpreted with caution given the small cohort sizes of both studies.

Previous studies suggesting immunotherapy directed at PD-1 as a suitable treatment option for glioblastoma are based on the presence of PD-L1 in tumour tissue [6, 7], and the success of anti-PD-1 in preclinical models [26, 39]. However, in order to assess PD-1, a number of prior studies such as those by Dejaegher et al (2017) [40] and Mohme et al (2018) [41], have used flow cytometry and reported a significant increase in PD-1 expression in T cells derived from glioblastoma tissue compared to peripheral blood. While flow cytometry has the benefit of being able to assess multiple markers on the same cell population, and can assess a larger number of cells than examination of tissue sections, it is not possible to know whether the cells of interest had indeed infiltrated the tumour tissue proper or were instead present in blood vessels, perivascular spaces, or as the result of haemorrhages, which were prominent in a number of cases in our study. Immunohistochemical analysis is considered to be the gold standard for assessing tumour infiltrating immune cells, as it accounts for localization and density of subsets of cells [42]. In agreement with previous immunohistochemical assessment of PD-1 [6, 8], our data demonstrate low density of PD-1+ TILs in only a minority of glioblastoma cases. If the current cohort is representative of glioblastoma cases, it is perhaps not surprising that anti-PD-1 checkpoint inhibition approaches have not shown significant survival benefit for patients.

Large numbers of preclinical rodent models of glioblastoma have assessed the effects of anti-PD-1 immune checkpoint inhibition alone [39] and in combination with various other treatments [26–28, 43], including radiation [26, 43] and temozolomide [43]. These studies have reported positive outcomes, including, but not limited to, long term-survival with single-agent anti-PD-1 [39], and survival enhanced by combination anti-PD-1 and radiation, which was attributed to increased cytotoxic CD8+ T cell infiltration [26]. Long term tumour-specific immune memory that prevented tumour development after tumour rechallenge has also been reported [26, 39]. While these studies use immunocompetent models of rodent glioma, suggesting they are relevant for assessing immune responses and immunotherapeutic approaches, rodent models have been reported to lack the diagnostically necessary histological characteristics that are seen in spontaneous human glioblastoma (i.e., palisading necrosis or microvascular proliferation) [44]. As rodent gliomas may not accurately represent human glioblastomas, response to anti-PD-1 treatment in these models may not be indicative of its clinical efficacy.

Glioblastomas are highly vascular with increased angiogenic activity associated with the presence of hemorrhaging [45] and increased presence of T cells [17]. Indeed, perivascular T cells were common in both primary and recurrent tumours in our cohort and Mu et al (2017) [46] found that the majority of T cells in both primary and recurrent glioblastoma were present in this location. However, perivascular T cells have also been reported in brain tissue without history of neurological or neuroinflammatory conditions [14], and in cohorts with neurological and neurodegenerative conditions [47]. Whether perivascular T cell density differs in glioblastoma and is associated with patient outcomes warrants investigation. We also observed CD3+ T cells in bleeds and associated with haemosiderin deposits, which indicate the occurrence of a previous haemorrhage. In cases with very prominent haemosiderin this accounted for the majority of T cells present in the tumour. It is therefore possible that T cells observed in association with bleeds have entered the tissue as a result of haemorrhage, as opposed to infiltrating the tumour as part of an immune response. This should be taken into consideration when designing studies using tumour tissue that has undergone dissolution to provide single cell suspensions for flow cytometric analysis of immune cell populations.

A limitation of our study is that it did not assess the relationship between TIL density and presence of tumour-associated macrophages. Tumour-associated macrophages including peripheral macrophages and resident microglia, are the major infiltrating immune cell population in primary [6, 48] and recurrent glioblastoma [49]. Tumour-associated macrophages are able to adopt tumour supportive, immunosuppressive phenotypes in response to stimuli from the surrounding environment, and therefore may impact TIL populations. For example, tumour-associated macrophages from glioblastoma have been reported to lack the costimulatory molecules CD86, CD80 and CD40, suggesting that they are not able to effectively support activation of T cells [48]. Future studies should incorporate assessment of macrophage/microglia populations in tissue from primary and recurrent glioblastoma.

Variability of PD-L1 labelling within and between tissue sections in this cohort also presented a limitation. The criteria relating to PD-L1 expression that determine suitability of treatment with anti-PD-1/anti-PD-L1 immunotherapies vary between tumours. For example, checkpoint inhibition is considered as a first line therapy in non-small cell lung cancer if greater than 50% of tumour cells demonstrate membranous PD-L1 labelling [50], while for urothelial carcinoma both the presence of immune cells in the tumour and the percentage of immune cells with positive PD-L1 labelling inform the decision to treat with immunotherapy [51]. Given that PD-L1 labelling occurred in multiple cell types and cellular localisations within and between tumours in our cohort (data not shown), determining the significance of each labelling pattern was not possible in a cohort of this size.

In summary, the level of T cell infiltration seen in this small cohort of matched primary and recurrent glioblastoma tissue was low. Though the number of CD8+ TILs was significantly higher in recurrent compared to primary tumours, overall TIL density at recurrence was still mild. PD-1+ TILs in particular were absent in the majority of our cases. Whether this is why many clinical trials using anti-PD-1 immunotherapy have not shown significant survival benefit in glioblastoma requires further investigation.

## MATERIALS AND METHODS

### Patient cohort

This study was approved by the Human Research Ethics Committee of the University of Newcastle (H-2018-0007). Formalin-fixed paraffin-embedded tumour tissue from maximal safe surgical resection was sourced from the Hunter Cancer Biobank (HCB) for 13 cases of glioblastoma with matched primary and recurrent tissue (Table 2). A block containing maximal tumour content was chosen from each patient and diagnosis of glioblastoma confirmed on H&E stained sections by a neuropathologist (REV). Clinical and demographic information available for the cohort included age, gender, location of primary tumour, *IDH1* mutation status, treatment information, and progression-free survival and overal survival information. *MGMT* promoter methylation status has also been reported where it was available in the clinical information. Ten of the 13 cases received standard treatment with radiation and temozolomide following the removal of the primary tumour. Complete treatment information was not available for two of the 13 cases (numbers 5 and 15), and one case (number 10) commenced radiation and temozolomide treatment following removal of the recurrent tumour (Table 2). Patients were considered as having had dexamethasone or temozolomide at the time of surgery if the last known date of drug treatment was within 28 days of the date of surgery. Only cases 9, 10 and 12 were known to have received dexamethasone and only case 9 was known to have received temozolomide within 28 days of recurrent surgery (Table 2). Additionally, the time between last dose of radiation and recurrent surgery was greater than 28 days for 10 of the 13 cases, unknown for two cases, and there was one case not treated with radiation prior to tumour recurrence.

**Table 2:** Demographic and clinical information available for glioblastoma cohort

Case number	Gender	Age at diagnosis	Primary tumour location	Treatment at primary	*IDH1* mutation status	*MGMT* promoter methylation	PFS (days)	OS (days)	Dexamethasone at surgery (days prior to surgery, dose)		TMZ at recurrent surgery (days prior to surgery)
									Primary	Recurrence	
2	M	39	Frontal lobe	RT/TMZ	WT	No	455	462^a^	No	No	No
3	F	77	Left inferolateral temporal lobe	RT/TMZ	WT	Unknown	938	1036	No	No	No
4	F	45	Left frontal lobe	RT/TMZ	WT	Unknown	398	679	No	No	No
5	M	76	Right frontal lobe	Unknown	WT	Unknown	315	316^a^	Unknown	Unknown	Unknown
6	M	72	Right temporoparietal lobe	RT/TMZ	WT	Unknown	266	343	No	No	No
7	M	42	Frontal lobe	RT/TMZ	WT	Unknown	374	453	No	No	No
8	F	81	Right temporal lobe	RT/TMZ	WT	Unknown	277	427	Unknown	Unknown	No
9	M	40	Anterior left temporal lobe	RT/TMZ	WT	Unknown	116	410	No	Yes (26 days^b^)	Yes (13 days)
10	M	55	Right frontal lobe	None	WT	No	38	177^a^	No	Yes (9 days, 1 mg)	No
11	M	72	Left temporal lobe	RT/TMZ	WT	Unknown	300	387	No	No	No
12	M	45	Left temporal lobe	RT/TMZ	WT	No	206	468	Unknown	Yes (2 days, 2 mg)	No
13	F	64	Right posterior temporoparietal lobe	RT/TMZ	WT	Unknown	252	919	No	No	No
15	F	65	Posterior right frontal lobe	Unknown	WT	Yes	310	353	Unknown	No	Unknown

Abbreviations: RT: radiation therapy; TMZ: temozolomide; *IDH1*: isocitrate dehydrogenase 1; WT: wild-type; *MGMT*: O-6-methylguanine-DNA methyltransferase; PFS: progression-free survival; OS: overall survival. ^a^Lost to follow-up. ^b^Started at 2 mg and weaned to 0 mg at 26 days prior to surgery.

### Immunohistochemistry

Formalin-fixed paraffin-embedded tumour tissue was sliced into 4 μm full face sections and processed for 3′,3′-diaminobenzidine (DAB) immunohistochemistry using a Ventana Discovery Ultra (Roche, USA) by the HCB. Sections were labelled for T cell markers CD3 (2GV6; Roche), CD4 (SP35; Roche), CD8 (SP57; Roche), and PD-1 (NAT105; Sigma-Aldrich, USA). All steps from baking to chromogen addition were performed automatically by the instrument. Tissue sections were baked to slides and deparaffinized, and antigen retrieval then occurred at 95°C/pH 9 with a total incubation time of 24 minutes prior to the addition of the primary antibody. Addition of the primary antibody was followed by a 32-minute incubation at 36°C. For CD4 labelling only, the primary antibody was dispensed before the blocking step, resulting in a reduction of non-specific labelling in the tumour tissue. Slides were then incubated with secondary antibody – Anti-Rabbit HQ for CD3, CD4 and CD8, and Anti-Mouse HQ (Roche) for PD-1 – for 20 minutes at 36°C. Positive control (normal tonsil) and negative control (normal brain) tissues were included in each batch of slides to confirm the specificity of antibody labelling (Supplementary Figure 2). Slides were digitally scanned using the Aperio^™^ Digital AT2 Pathology System (Leica Biosystems, Australia) for analysis.

### Categorical TIL scoring

The scoring system used for CD3, CD4, CD8 and PD-1 labelled tissue was developed in consultation with an experienced neuropathologist (REV) and based on the association of positive cells with characteristic topographical features of glioblastoma: tumour proper, perivascular areas (i.e., closely associated with blood vessels), and bleeds/haemorrhages (Figure 1). Signs of haemorrhage included presence of red blood cells or haemosiderin deposits within the tumour tissue (Figure 1). Digitised slides were assessed by scanning across the whole tissue section at 40× absolute magnification, with higher magnifications of 100–200× used as necessary to confirm T cell labelling or topographical features of the tissue. CD3+, CD4+, CD8+, and PD-1+ cells in each topographical area were given a density score of 0 – absent, 1 – present, 2 – moderate, or 3 – marked (Figure 2). Scoring was performed independently by two investigators (KM and PAT), and discrepancies were discussed and adjusted to determine final scores for each tissue section.

### Quantitative TIL scoring

HALO image analysis software (version 1; Indica Labs, USA) was used to quantify CD3+ and CD8+ TILs in the tumour proper of tissue sections labelled by immunohistochemistry. Based on CD3+ labelling, the software was trained to recognise DAB labelled T cells as positive, and to count these cells using a modified version of the inbuilt CytoNuclear analysis algorithm. Analysis parameters were adjusted until HALO could recognise and quantify DAB labelled cells accurately across a number of regions representative of TIL infiltration within and between tissue sections.

Whole tissue sections labelled for CD3 were examined within the HALO software at 40× magnification and four areas of highest CD3+ cell density within the tumour proper were identified. An annotation region of 245 × 245 μm (~60,000 μm^2^) was placed in each of the four hotspot areas and the number of CD3+ cells within each annotation quantified by HALO. Annotation regions were placed at least 245 μm from: other annotations, large blood vessels, necrotic tissue, haemorrhages, any “normal” tissue, and the edges of the tissue section. The mean density of TILs was determined based on positive cell counts within the four annotation regions for each tissue section. This process was repeated for CD8, CD4 and PD-1 labelled slides, placing annotations in the same hotspot regions as for the CD3 labelled sections. HALO was unable to accurately identify CD4+ and PD-1+ cells due to lower DAB intensity in CD4+ and PD-1+ cells and the presence of CD4 labelling in additional cell types. Positively labelled CD4+ and PD-1+ cells were therefore identified based on morphology and counted manually in each 245 × 245 μm annotated region.

### Statistical analysis

Two-tailed Wilcoxon matched pairs signed rank tests were used for all paired datasets to determine whether TIL density differed between primary and recurrent glioblastomas. Statistical analyses were performed using Prism software (version 8.1.2 GraphPad Software, USA) or SPSS (version 25, IBM Corporation, USA) and *p* < 0.05 was considered significant.

## SUPPLEMENTARY MATERIALS



## Abbreviations

CNS: Central nervous system
DAB: 3’,3’-diaminobenzidine
PD-1: programmed cell death-1
PD-L1: programmed cell death ligand 1
TILs: tumour-infiltrating lymphocytes
