# Supplementary material for: Low tumour-infiltrating lymphocyte density in primary and recurrent glioblastoma
Source: Oncotarget. 2021 Oct 12;12(21):2177–87. doi: 10.18632/oncotarget.28069 (PMC8522837; doi:10.18632/oncotarget.28069)
Supplement: Supplementary file 1 [file oncotarget-12-2177-s001.pdf]

Low tumour-infiltrating lymphocyte density in primary and recurrent glioblastoma

SUPPLEMENTARY MATERIALS

Supplementary Table 1: Spearman's Rho nonparametric correlation of categorical and numerical TIL scores in glioblastoma tissue

	Mean CD3 count/mm²		Mean CD8 count/mm²		Mean CD4 count/mm²		Mean PD-1 count/mm²	
	Spearman's r_s	*p*-value	Spearman's r_s	*p*-value	Spearman's r_s	*p*-value	Spearman's r_s	*p*-value
**CD3 score, tumour proper**	0.658	0.261×10^{-3}						
**CD8 score, tumour proper**			0.552	0.003				
**CD4 score, tumour proper**					0.697	0.756×10^{-4}		
**PD-1 score, tumour proper**							0.437	0.042

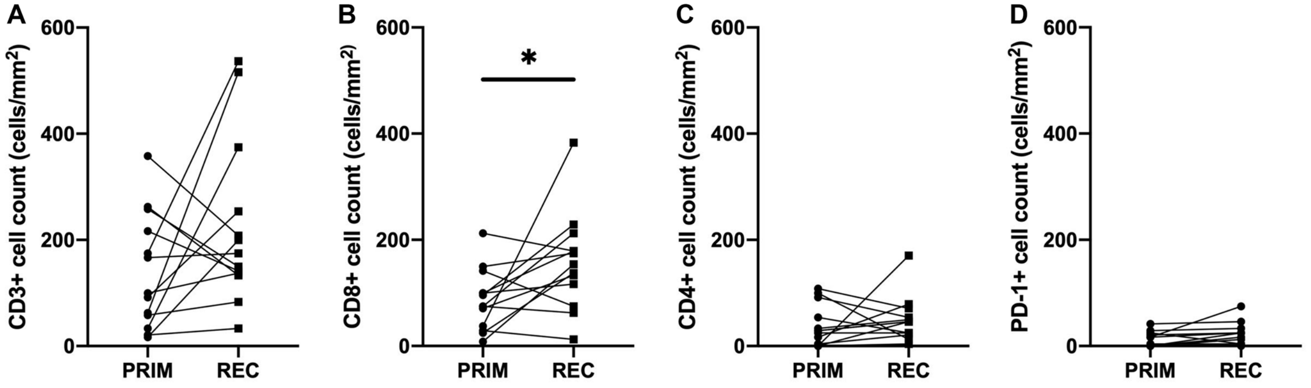

Supplementary Figure 1: Quantitative TIL scores in primary and recurrent glioblastoma. Density of CD3+, CD8+, CD4+ and PD-1+ TILs was calculated for each case and compared between primary and recurrent groups. No difference was seen in overall CD3+ TIL density (p =0 .191; (**A**) CD8+ TILs were present at significantly higher density in recurrent tumours compared to primary tumours ($p = 0.040$; (**B**) with 9 of 13 cases showing this trend in the recurrent tumour. No differences were seen in CD4+ TIL density ($p = 0.607$; (**C**) or PD-1+ TIL density ($p = 0.070$; (**D**) Bars link TIL density for the primary and recurrence from the same case. Abbreviations: PRIM: primary tumour; REC: recurrent tumour.

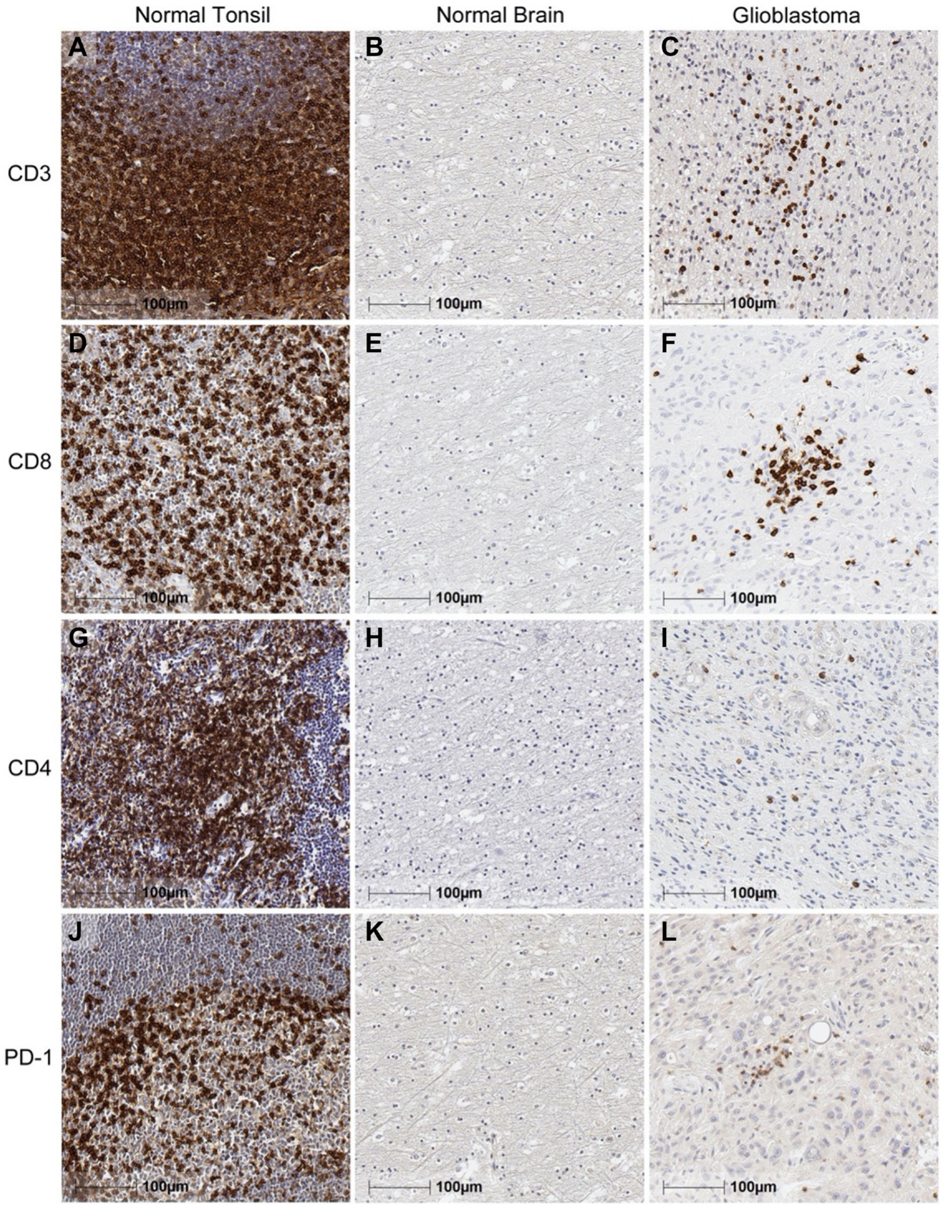

Supplementary Figure 2: Confirmation of specific labelling by CD3, CD4, CD8 and PD-1 antibodies. Glioblastoma tissue sections immunohistochemically labelled for CD3, CD4, CD8 and PD-1 were compared to positive and negative control tissue from the same labelling batch to confirm the specificity of antibody labelling. Normal tonsil tissue was used as the positive control, and demonstrated areas of dark brown positive labelling, for CD3+ (**A**), CD8+ (**D**), CD4+ (**G**), and PD-1+ (**J**) T cells. Normal brain tissue was used as a negative control and showed no positive labelling by any of the antibodies (**B**, **E**, **H**, **K**). Specific CD3+ (**C**), CD8+ (**F**), CD4+ (**I**), and PD-1+ (**L**) labelling of TILs was observed in glioblastoma tissue.
